# Endocrine Adverse Events Induced by Cancer Treatments: The Role of ^18^F-Fluorodeoxyglucose Positron Emission Tomography

**DOI:** 10.3390/cancers17162651

**Published:** 2025-08-14

**Authors:** Luca Giovanella, Murat Tuncel, Alfredo Campennì, Rosaria Maddalena Ruggeri, Martin Huellner, Petra Petranović Ovčariček

**Affiliations:** 1 Department of Nuclear Medicine and Thyroid Diseases, Gruppo Ospedaliero Moncucco, 6900 Lugano, Switzerland; 2 Department of Nuclear Medicine and Thyroid Centre, University Hospital of Zurich, 8091 Zurich, Switzerland; 3 Department of Clinical Chemistry and Endocrinology, Medysin, 6004 Luzern, Switzerland; 4 Department of Nuclear Medicine, Hacettepe University, 06100 Ankara, Turkey; murat.tuncel@hacettepe.edu.tr; 5 Unit of Nuclear Medicine, Department of Biomedical and Dental Sciences and Morpho-Functional Imaging, University of Messina, 98124 Messina, Italy; 6 Unit of Endocrinology, Department of Clinical and Experimental Medicine, University of Messina, 98124 Messina, Italy; rosaria.ruggeri@unime.it; 7 Department of Nuclear Medicine, University Hospital Zurich, University of Zurich, 8091 Zurich, Switzerland; martin.huellner@usz.ch; 8 Department of Oncology and Nuclear Medicine, University Hospital Center Sestre Milosrdnice, 10000 Zagreb, Croatia; p.petranovic@gmail.com; 9 School of Medicine, University of Zagreb, 10000 Zagreb, Croatia

**Keywords:** PET/CT imaging, FDG, immune-related adverse events, endocrine toxicity, metabolic imaging, cancer immunotherapy, thyroiditis, pancreatitis, hypophysitis, adrenalitis, precision oncology

## Abstract

Immune checkpoint inhibitors (ICIs) and tyrosine kinase inhibitors (TKIs) have revolutionized cancer therapy, substantially improving survival across a broad range of malignancies. However, these agents are associated with a unique profile of endocrine immune-related adverse events (irAEs), including thyroiditis, hypophysitis, adrenalitis, and pancreatitis, which differ significantly from the toxicities seen with conventional chemotherapy. These complications often arise unpredictably during treatment and may result in irreversible hormone deficiencies requiring lifelong replacement, underscoring the importance of early detection. ^18^F-fluorodeoxyglucose positron emission tomography/computed tomography (^18^F-FDG PET/CT) has emerged as a valuable tool not only for oncologic staging and response assessment but also for detecting metabolic changes in endocrine organs. PET/CT can identify irAEs before the appearance of clinical symptoms or biochemical abnormalities. Emerging evidence suggests that the presence of endocrine irAEs identified by ^18^F-FDG PET/CT may correlate with improved treatment response and survival, possibly reflecting enhanced immune activation. This comprehensive review discusses the role of ^18^F-FDG PET/CT in the early recognition of therapy-induced endocrine toxicities, facilitating timely intervention through hormone replacement or immunosuppressive therapy while minimizing unnecessary treatment interruptions. Effective integration of metabolic imaging with clinical and laboratory evaluation requires coordinated multidisciplinary collaboration among oncologists, endocrinologists, and nuclear medicine physicians to optimize outcomes and reduce endocrine-related morbidity in the era of precision oncology.

## Simple Summary

Novel cancer therapies, particularly immune checkpoint inhibitors and targeted agents, have significantly improved survival rates for many patients. However, these treatments may lead to adverse effects in hormone-producing glands such as the thyroid, pituitary, and adrenal glands. These endocrine disturbances can markedly impact quality of life and may require lifelong hormone replacement therapy. This review explores the role of ^18^F-fluorodeoxyglucose positron emission tomography/computed tomography (^18^F-FDG PET/CT) in the early detection of endocrine-related toxicities. This imaging modality can reveal increased metabolic activity in affected glands, often before the onset of symptoms or laboratory abnormalities, enabling prompt initiation of appropriate treatment. Early identification of these complications is critical to allow effective management without interrupting cancer therapy. Optimal care requires close collaboration between oncologists, endocrinologists, and nuclear medicine specialists to minimize treatment-related morbidity while maintaining therapeutic efficacy.

## 1. Introduction

The therapeutic landscape of oncology has been fundamentally transformed by the advent of chemotherapy, advanced radiotherapy techniques, targeted therapies, and immunotherapy. Among these, tyrosine kinase inhibitors (TKIs) and immune checkpoint inhibitors (ICIs) have markedly improved survival outcomes across a broad spectrum of malignancies. However, these advances have also introduced a distinct spectrum of adverse effects (AEs) that diverge from the classical toxicities associated with conventional chemotherapy and radiotherapy. While cytotoxic agents typically induce dose-dependent myelosuppression, gastrointestinal toxicity, and mucositis, TKIs are frequently related to off-target toxicities, including endocrine, cardiovascular, and dermatologic complications, due to their effects on signaling pathways in non-malignant tissues. Similarly, ICIs, by modulating immune tolerance, can trigger a wide range of immune-related adverse events (irAEs), which may affect virtually any organ system. Among the most clinically significant irAEs are endocrinopathies, including thyroiditis, hypophysitis, adrenalitis, and, less commonly, pancreatitis, emphasizing the need for vigilant monitoring and specialized management strategies [1]. Endocrine adverse effects (EAEs) are generally less common than the hematologic, gastrointestinal, and dermatologic toxicities seen with cytotoxic chemotherapy, where hormonal dysfunction is usually limited to gonadal suppression or thyroid abnormalities associated with select regimens. Radiotherapy (RT), on the other hand, primarily causes localized toxicities, with the nature of AEs determined by the irradiated anatomical region. Common RT-induced complications include skin reactions, mucositis, and fibrosis. Nevertheless, EAEs remain relevant, particularly when treatment fields involve the head, neck, mediastinum, or pelvis, where radiation exposure may lead to thyroid dysfunction, hypopituitarism, or gonadal failure, respectively. For instance, hypothyroidism occurs in up to 50% of patients undergoing neck irradiation, and hypopituitarism develops in over 30% of patients receiving cranial radiotherapy involving the hypothalamic–pituitary axis, often presenting years after treatment completion [2,3,4,5]. Although EAEs are typically less frequent and slower to manifest than acute mucosal or hematologic toxicities, they often result in irreversible hormone deficiencies, with a substantial impact on the long-term quality of life in cancer survivors. Therefore, heightened clinical awareness and structured long-term surveillance are essential components of survivorship care in modern oncology. TKIs are well-recognized for inducing thyroid dysfunction, reported in up to 50% of treated patients [6]. These effects are frequently subclinical, but their persistence or progression warrants endocrinological evaluation. In contrast, the advent of ICIs has fundamentally altered the toxicity profile in cancer therapy. Endocrine irAEs are now among the most common and clinically relevant complications, including thyroiditis (6–34%), hypophysitis (3–14%), and adrenalitis (0.7–1.6%) [7]. Importantly, these toxicities often result in permanent endocrine insufficiencies, necessitating lifelong hormone replacement therapy. This reality underscores the necessity for routine endocrine surveillance in patients receiving ICIs and TKIs [8,9,10,11]. The unpredictable nature of EAEs, especially irAEs, demands specific monitoring protocols, including targeted endocrine testing and, when indicated, immunosuppressive therapy. Early recognition and appropriate management are critical to prevent severe endocrine crises and to maintain continuity of effective cancer treatment. Crucially, metabolic and molecular imaging, particularly ^18^F-fluorodeoxyglucose positron emission tomography/computed tomography (^18^F-FDG PET/CT), has emerged as a non-invasive tool capable of detecting subclinical endocrine inflammation. PET/CT may identify increased metabolic activity in affected glands before the onset of clinical symptoms or biochemical abnormalities. This allows for timely intervention, reduces the risk of complications, and supports uninterrupted oncologic therapy. This review aims to highlight the expanding role of ^18^F-FDG PET/CT in the detection and management of endocrine adverse effects associated with TKIs and ICIs, emphasizing the need for multidisciplinary collaboration in the era of precision oncology.

## 2. Treatment-Induced Adverse Effects in Oncological Patients

### Prevalence, Pathogenesis, and Clinical Presentation

Relevant endocrine side effects associated with different oncological treatments, along with their prevalence across different therapeutic approaches and cancer types, are summarized in Table 1 and Table 2.

Chemotherapy remains a cornerstone of oncologic treatment, exerting its therapeutic effect by targeting rapidly dividing cells. However, this non-selective mechanism also underlies a well-characterized spectrum of predictable, dose-dependent adverse effects. The most common toxicities include myelosuppression, leading to neutropenia, anemia, and thrombocytopenia, which subsequently increase the risk of infection and bleeding. Gastrointestinal toxicity, such as nausea, vomiting, mucositis, and diarrhea, results from damage to the rapidly renewing gastrointestinal epithelium. Alopecia, reflecting the vulnerability of hair follicles to cytotoxic agents, is another frequent side effect. In addition, specific chemotherapeutic agents are associated with organ-specific toxicities—including cardiotoxicity with anthracyclines, neurotoxicity with platinum-based agents and taxanes, and nephrotoxicity with cisplatin. These risks necessitate close monitoring and, when appropriate, dose modifications to minimize long-term harm. Endocrine adverse effects (EAEs), while less common with conventional chemotherapy, primarily involve gonadal suppression [12]. Hormone therapies, including aromatase inhibitors used in breast cancer and androgen-deprivation therapy (ADT) in prostate cancer, often lead to profound hypogonadism, with associated risks of osteoporosis and metabolic disturbances [13]. Radiation therapy directed at the brain, neck, or pelvis is also related to delayed-onset endocrine dysfunction. Cranial irradiation may lead to deficiencies in growth hormone (GH), thyroid-stimulating hormone (TSH), and adrenocorticotropic hormone (ACTH). Neck irradiation commonly results in hypothyroidism, while pelvic radiation frequently causes gonadal failure, particularly in premenopausal women and children [14]. As previously noted, the introduction of molecular targeted therapies and immune checkpoint inhibitors (ICIs) has markedly increased the incidence and clinical relevance of EAEs [15]. For example, tyrosine kinase inhibitors (TKIs) such as sunitinib, widely used in renal cell carcinoma, are associated with hypothyroidism in up to 70% of patients [16]. Similarly, mammalian target of rapamycin (mTOR) inhibitors, employed in the treatment of hepatic and pancreatic cancers, are frequently associated with hyperglycemia and insulin resistance, necessitating regular metabolic monitoring [17]. Immune checkpoint inhibitors (ICIs) are monoclonal antibodies that target regulatory cell surface molecules (e.g., CTLA-4, PD-1, PD-L1) on immune cells, thereby releasing the body’s natural immunologic “brakes” and promoting immune-mediated tumor destruction. In aggressive malignancies such as malignant melanoma and non-small cell lung cancer, ICIs have led to significant improvements in overall survival. However, their unique immune-mediated toxicity profile differs substantially from those of traditional therapies and can affect multiple non-target organs, including endocrine glands [18]. Specifically, ICIs are associated with a significantly increased risk of thyroid dysfunction, adrenalitis, hypophysitis, and immune-related diabetes [19]. A meta-analysis of 28 clinical trials demonstrated that the incorporation of ICIs in perioperative regimens was associated with higher rates of grade 3–4 treatment-related adverse events and increased treatment discontinuation [20]. Although adrenal insufficiency and immune-related diabetes are less common, they remain clinically significant and challenging to manage. Endocrine irAEs may present early, shortly after the initiation of treatment, or may emerge later, even after therapy has been discontinued [21]. These events are more frequently observed in patients with lung cancer and melanoma, and typically manifest within the first few weeks to months of therapy [22]. While the majority of cases are mild (grade 1–2), rare but severe complications—such as adrenal crisis or severe thyrotoxicosis—may necessitate temporary treatment interruption or discontinuation [23]. Fortunately, hormone imbalances—even in critical cases—can often be rapidly corrected, minimizing disruption to ongoing oncologic therapy. Nonetheless, it is essential to note that endocrinopathies induced by TKIs or ICIs may result in chronic organ damage and permanent hormonal insufficiency, requiring lifelong hormone replacement therapy [18].

## 3. Clinical Management and PET/CT Imaging

Differentiating the symptoms and signs of endocrinopathies from those caused by the underlying malignancy or other oncologic treatments can be challenging. Many symptoms of endocrine dysfunction—such as fatigue, weakness, increased sweating, tachycardia, weight changes, polydipsia, nausea, vomiting, and abdominal pain—are nonspecific and frequently overlap with those experienced by cancer patients during therapy. Consequently, personalized surveillance is essential during both active treatment and the survivorship phase. Routine biochemical monitoring of thyroid, adrenal, and gonadal function enables early diagnosis of endocrine adverse events (EAEs), thereby contributing to improved clinical outcomes [21,24]. Importantly, pre-existing and well-controlled endocrinopathies are not contraindications to initiating or continuing immune checkpoint inhibitors (ICIs) or other oncologic therapies. The benefit-to-risk ratio remains favorable, and EAEs are typically manageable with appropriate supportive care [19]. For instance, in a cohort of 143 patients with metastatic melanoma and pre-existing autoimmune endocrine disease, the incidence of grade ≥3 immune-related adverse events (irAEs) was comparable to that in patients without pre-existing autoimmune conditions. Treatment responses were also similar between the groups [25]. Newly diagnosed endocrine dysfunctions that arise during cancer therapy rarely necessitate dose reductions or treatment discontinuation. Instead, these cases should be evaluated and managed within a multidisciplinary team, involving oncologists, endocrinologists, and other specialists, to ensure optimal outcomes while preserving the efficacy of cancer therapy [26]. Effective management of EAEs hinges on comprehensive pretreatment evaluation, ongoing monitoring, and timely intervention. This process begins with a thorough clinical assessment and baseline hormone measurements, followed by periodic reassessment to detect subclinical endocrine dysfunctions and initiate hormone replacement therapy or other treatments as needed.

While anatomical imaging modalities such as computed tomography (CT), magnetic resonance imaging (MRI), and ultrasound (US) are routinely employed to assess tumor burden and structural changes in endocrine glands, these techniques may fail to capture early metabolic or functional abnormalities. In contrast, molecular imaging, particularly ^18^F-fluorodeoxyglucose positron emission tomography/computed tomography (^18^F-FDG PET/CT), enables the visualization of tissue metabolism and cellular activity, offering a sensitive means of detecting subclinical or evolving toxicities. This provides an opportunity for early therapeutic intervention. Beyond its well-established role in cancer staging and response assessment, ^18^F-FDG PET/CT has demonstrated utility in detecting treatment-related complications, including chemotherapy-induced cardiotoxicity [27,28] and radiation-induced pulmonary and bone marrow injury [29,30]. As the landscape of oncologic treatment evolves, PET/CT has become increasingly valuable in identifying adverse effects associated with TKIs and ICIs. These novel therapies, designed to target tumor-specific signaling or enhance immune responses, can inadvertently affect normal biological processes, resulting in a spectrum of cell-specific, organ-specific, or systemic toxicities. Many of these are mediated by immune activation or microvascular dysfunction, often accompanied by increased glucose metabolism, which is readily visualized on ^18^F-FDG PET/CT. Increased ^18^F-FDG uptake in endocrine glands is not restricted to EAEs, and different potential causes must be well understood by the nuclear medicine physician in order to perform an accurate differential diagnosis (Table 3).

Notably, treatment-related metabolic changes can mimic disease progression on imaging or co-exist with actual tumor progression. One well-documented phenomenon is immunotherapy-induced pseudo progression, characterized by an initial increase in lesion size or appearance of new hypermetabolic foci on imaging, representing immune cell infiltration and inflammatory changes rather than tumor growth. This is typically followed by subsequent tumor regression [31,32]. On PET/CT, pseudo progression presents as increased FDG uptake in pre-existing lesions or as new sites of hypermetabolism, which may confound response assessment. Accurate differentiation between pseudo progression and actual progression is crucial to avoid unwarranted treatment discontinuation. To support this need, dedicated response criteria—including PERCIMT (PET Response Criteria in Immunotherapy) and iPERCIST—have been developed to guide interpretation of metabolic changes during immunotherapy [32]. When progression is suspected, serial PET/CT imaging and assessment of metabolic trends over time are recommended rather than relying on a single scan. Additionally, recognition of FDG uptake patterns associated with irAEs, such as diffuse thyroid uptake or increased pituitary activity, can offer valuable diagnostic context [33]. Identifying these findings within the clinical framework of immunotherapy can inform appropriate treatment decisions and help prevent premature cessation of effective therapies. Among the most common endocrine irAEs identifiable by PET/CT are thyroiditis, hypophysitis, adrenalitis, and pancreatitis. These conditions often present as abnormal diffuse or, rarely, focal FDG uptake in the affected glands, prompting further diagnostic evaluation and, when necessary, initiation of glucocorticoid therapy or other immunosuppressive treatments. Sequential PET/CT imaging, primarily performed to monitor cancer response to therapy, may also provide information concerning the resolution or persistence of active inflammation within endocrine glands. Other diseases can be related to FDG uptake within endocrine glands (Table 4). Still, it should be considered that FDG patterns of EAEs largely overlap those of primary autoimmune endocrine diseases, sharing multiple etiological and pathogenetic aspects with the latter.

In the following sections, we examine the clinical manifestations, diagnostic strategies, and management approaches for specific therapy-induced endocrinopathies, with a particular focus on the contribution of ^18^F-FDG PET/CT imaging to early detection and treatment guidance.

### 3.1. Thyroid Adverse Effects

There is growing evidence that targeted cancer therapies—including tyrosine kinase inhibitors (TKIs) and immune checkpoint inhibitors (ICIs)—have a significant impact on thyroid function, placing a substantial proportion of patients at risk for developing various thyroid disorders [6,34]. These dysfunctions may range from subclinical to overt hypothyroidism or thyrotoxicosis and frequently arise within the first few weeks to months of treatment. However, particularly in the context of ICIs, thyroid dysfunction can also emerge unpredictably, at any point during therapy or even after its completion [18].

**Figure 1:**
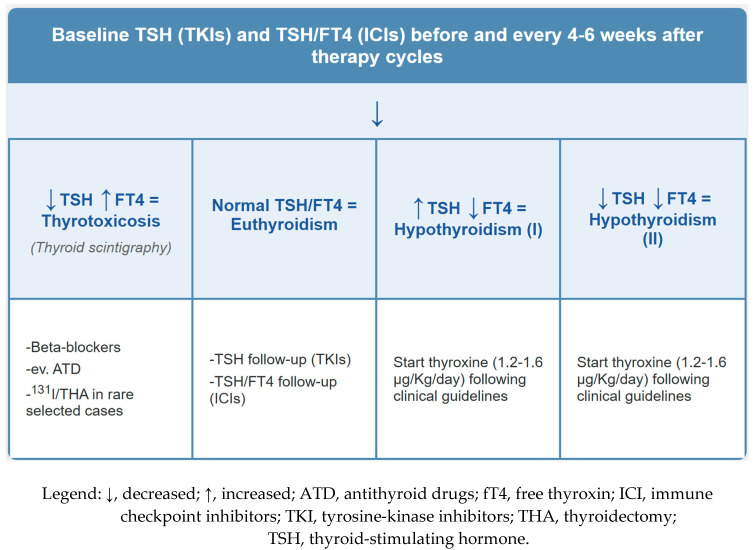
Screening, diagnostic, and clinical actions for TKIs/ICIs-induced thyroid diseases.

A meta-analysis of 12 clinical trials confirmed a significantly increased risk of all-grade hypothyroidism in patients treated with TKIs [35]. The mechanisms underlying TKI-induced hypothyroidism are multifactorial and may include destructive thyroiditis due to vascular damage, ischemic injury, and eventual thyroid gland atrophy. Additionally, TKIs are known to increase levothyroxine requirements in thyroidectomized patients—likely due to extra-thyroidal mechanisms such as altered gastrointestinal absorption and enterohepatic circulation of thyroid hormone [6,36]. ICIs have also been strongly associated with thyroid dysfunction. In a meta-analysis of 38 clinical trials involving 7551 patients, combination ICI therapy conferred a significantly higher risk of thyroid dysfunction compared to monotherapy [37]. Both TKIs and ICIs commonly induce primary hypothyroidism, which is frequently preceded by a transient thyrotoxic phase. This biphasic pattern occurs in up to 40% of TKI-treated patients and up to 80% of those receiving ICIs. Graves’ disease, although rare, has been reported predominantly in association with ICIs [6]. Thyrotoxic symptoms—including tachycardia, tremor, anxiety, and insomnia—are generally mild and transient. However, late-onset hypothyroidism develops in a substantial proportion of patients and often necessitates long-term thyroxine replacement therapy. While thyroid imaging is not routinely indicated, thyroid scintigraphy can be valuable for distinguishing destructive thyroiditis (characterized by low radiotracer uptake) from Graves’ disease (characterized by increased uptake), thereby guiding appropriate therapeutic interventions [6]. Crucially, the nonspecific nature of symptoms associated with thyroid dysfunction—particularly fatigue and weight changes—can lead to misattribution to the underlying cancer or other treatments, potentially resulting in delayed diagnosis and unnecessary modifications of oncologic therapy [6,38]. Fortunately, heightened awareness among oncologists, endocrinologists, and nuclear medicine physicians has led to the establishment of evidence-based screening and monitoring protocols for thyroid dysfunction during TKI and ICI therapy [6,39]. Assessment of thyroid-stimulating hormone (TSH) remains the primary screening test. If TSH is abnormal, a reflex-free thyroxine (fT4) measurement is recommended. Thyroid dysfunction is highly unlikely when TSH remains within the normal range. However, special consideration is required in patients receiving ICIs—particularly anti-CTLA-4 agents—as these may induce hypophysitis, resulting in central hypothyroidism. In such cases, TSH levels may appear normal or low, despite reduced fT4 and the presence of clinical signs of hypothyroidism. Thus, simultaneous measurement of TSH and fT4 is recommended in patients receiving ICIs, rather than relying solely on a TSH-reflex testing strategy [6]. Baseline thyroid function testing is advised before the initiation of TKI or ICI therapy, followed by periodic monitoring every 4–6 weeks during treatment. An evidence-based diagnostic and clinical management algorithm for TKI/ICI-induced thyroid dysfunction is presented in Figure 1.

**Figure 2:**
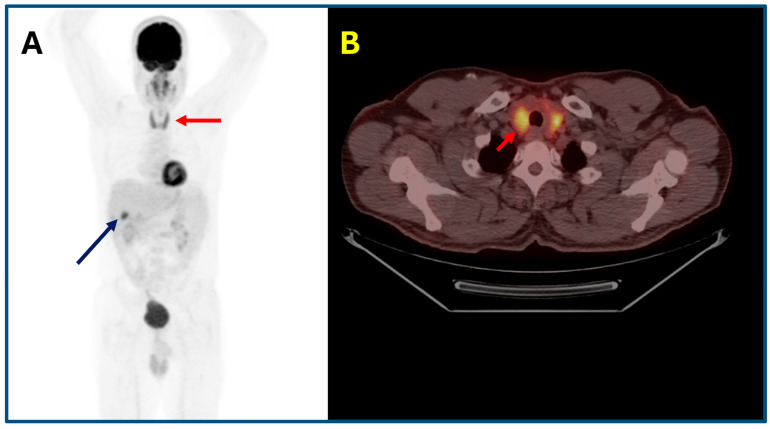
Immune-related thyroiditis in a 67-year-old male patient with colorectal cancer receiving Pembrolizumab at a dose of 2 mg/kg IV every 3 weeks for metastatic disease noted in the liver. PET maximum intensity projection (MIP) (**A**) and axial PET/CT fusion images (**B**) showed a diffuse increase in ^18^F-FDG uptake within the thyroid gland (red arrow) and ^18^F-FDG active liver metastasis (dark blue arrow).

Diffuse ^18^F-FDG uptake in the thyroid gland is a frequently observed immune-related adverse event (irAE) in patients receiving immune checkpoint inhibitors (ICIs), reported in approximately 6–34% of cases, depending on the therapeutic regimen and timing of imaging. Importantly, ^18^F-FDG PET/CT is often able to detect “isolated metabolic thyroiditis” at a subclinical stage, preceding biochemical abnormalities and clinical symptoms. This imaging-based finding may serve as an early warning sign of evolving thyroid dysfunction. For instance, in a cohort of melanoma patients treated with combined ipilimumab and nivolumab, 34% developed thyroiditis within three weeks of treatment initiation, and ^18^F-FDG uptake predicted thyroiditis with a positive predictive value of 93% [40]. Additionally, a recent study employing AI-based segmentation techniques demonstrated that an increase in thyroid SUVmax following ICI initiation was strongly associated with clinically manifest thyroid dysfunction. Specifically, patients with elevated SUVmax had 13.8-fold increased odds of developing thyroid adverse events [41]. These findings emphasize that incidental diffuse thyroid ^18^F-FDG uptake on PET/CT functions not only as a sensitive biomarker for immune-mediated thyroiditis but also as an early predictive marker, guiding endocrine surveillance and facilitating timely initiation of hormone replacement therapy (Figure 2).

### 3.2. Immunotherapy-Induced Pituitary Diseases

**Figure 3:**
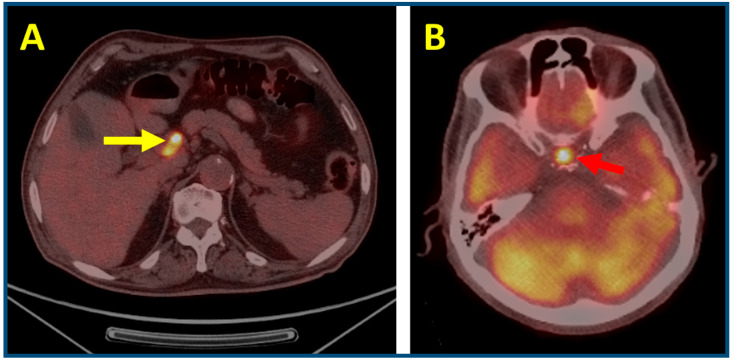
A 65-year-old man with melanoma metastatic to abdominal lymph nodes ((**A**); axial PET/CT fusion images, yellow arrow) underwent a combination of ipilimumab and nivolumab. Following treatment initiation, an ^18^F-FDG PET/CT scan was performed to assess response. A focal pituitary uptake, highly suspicious for hypophysitis, was detected ((**B**); axial PET/CT fusion images, red arrow). Although the patient was asymptomatic, biochemical evaluation confirmed panhypopituitarism, and appropriate endocrine therapy was started.

Immune checkpoint inhibitors (ICIs), particularly anti-CTLA-4 agents or combined CTLA-4/PD-1 regimens, are associated with an increased risk of hypophysitis, occurring in approximately 3–14% of treated patients. This immune-related adverse event (irAE) often presents with nonspecific symptoms, making early diagnosis challenging. Hypophysitis is most frequently associated with CTLA-4 inhibitors, either alone or in combination with PD-1/PD-L1 blockade. It requires prompt recognition and structured management to avoid potentially life-threatening complications, including secondary adrenal insufficiency and other pituitary hormone deficiencies. Upon clinical suspicion, a comprehensive endocrine evaluation should be initiated, including measurement of serum cortisol, ACTH, TSH, free T4, prolactin, LH, FSH, and electrolytes. Additionally, pituitary MRI should be performed to exclude metastases, mass lesions, or other structural abnormalities. While high-dose corticosteroids (e.g., prednisone 0.5–1 mg/kg) may be warranted in cases with severe headache or pituitary enlargement, the cornerstone of management in most cases is targeted hormone replacement therapy, as hypophysitis frequently results in irreversible hormone deficiencies. Glucocorticoid replacement (e.g., hydrocortisone 15–25 mg/day) should be initiated promptly in cases of adrenal insufficiency, with stress dosing during intercurrent illness or surgery to prevent adrenal crisis. Importantly, thyroid hormone replacement should only be introduced after cortisol deficiency has been addressed to avoid precipitating adrenal decompensation. While corticosteroids may reduce inflammation and symptoms, they do not typically restore endocrine function and may attenuate the therapeutic efficacy of immunotherapy; thus, the decision to initiate high-dose steroids must be based on an individualized risk–benefit analysis. Immunotherapy is usually temporarily withheld during acute endocrine management but can be safely resumed once the patient stabilizes, especially in the absence of significant mass effect. Long-term endocrine follow-up is essential, as most patients develop lifelong hormone deficiencies and may require ongoing surveillance for additional pituitary dysfunction, supporting survivorship care planning. Recently, ^18^F-FDG PET/CT has emerged as a valuable adjunct in the early detection of hypophysitis, particularly when MRI findings are inconclusive or nonspecific. A case-control study in patients with metastatic melanoma demonstrated significantly elevated pituitary-to-blood pool target-to-background ratios (TBRs) in individuals with confirmed hypophysitis compared to controls (median TBR ≈ 2.78 vs. 1.59). Using a TBR threshold of ~2.4, PET imaging achieved 72.7% sensitivity and 90.9% specificity for the early diagnosis of hypophysitis [33]. Furthermore, a prospective cohort study involving 162 patients receiving ipilimumab plus nivolumab reported a median 63% increase in pituitary SUVmax following treatment. Significantly, new focal ^18^F-FDG uptake preceded clinical diagnosis by a median of 16 days. Receiver operating characteristic (ROC) analysis indicated that a ≥ 30% increase in SUVmax reliably predicted the development of hypophysitis [42]. These findings highlight that quantitative PET parameters, such as TBR and changes in SUVmax, may serve as early metabolic biomarkers of immunotherapy-induced hypophysitis, enabling timely endocrine evaluation and intervention before critical hormone deficiencies manifest. Integration of PET/CT findings into routine surveillance may thus support proactive clinical management and improve patient outcomes (Figure 3).

### 3.3. Immunotherapy-Induced Adrenal Diseases

Adrenalitis is a rare but clinically significant immune-related adverse event (irAE) associated with immune checkpoint inhibitor (ICI) therapy, particularly anti-CTLA-4 agents. The incidence of primary adrenal insufficiency (PAI) among patients treated with ICIs ranges from approximately 0.7% to 1.6%, though it may be underestimated due to nonspecific clinical presentations. Key presenting symptoms include fatigue, hypotension, nausea, salt craving, and unintentional weight loss, which can easily be mistaken for general cancer-related symptoms or treatment-related side effects. Initial evaluation should include electrolyte assessment, as hyponatremia and hyperkalemia may suggest adrenal insufficiency. Measurement of early morning serum cortisol and ACTH is essential, and if results are equivocal, a standard ACTH stimulation test (cosyntropin test) should be performed to confirm the diagnosis. Upon confirmation or strong suspicion of adrenal insufficiency, glucocorticoid replacement therapy with hydrocortisone (15–25 mg/day in divided doses) should be initiated promptly. If there is evidence of mineralocorticoid deficiency, which is typical of primary (rather than central) adrenal insufficiency, fludrocortisone (0.05–0.1 mg/day) should also be administered. High-dose corticosteroids are generally not required unless there is suspicion of severe inflammation with systemic involvement. Temporary interruption of immunotherapy may be considered in hemodynamically unstable patients; however, ICI treatment can often continue safely with appropriate hormone replacement.

**Figure 4:**
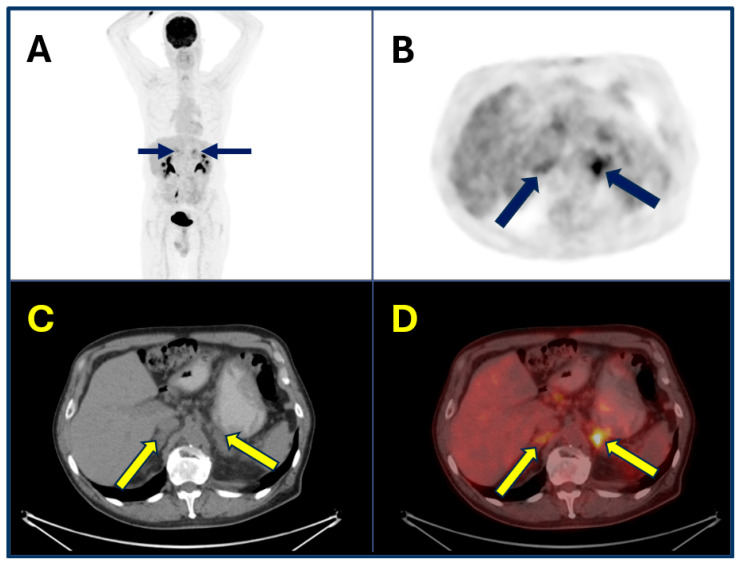
A 49-year-old man with metastasized melanoma received immunotherapy with ipilimumab and nivolumab. Six months after therapy initiation, FDG-PET/CT [(**A**,**B**), (**A**): maximum intensity projection; (**B**): axial attenuation corrected section; (**C**,**D**), CT and fused PET/CT axial images] revealed slightly enlarged and ^18^F-FDG-avid adrenal glands ((**A**,**B**): blue arrows; (**C**,**D**): yellow arrows). Subsequent ACTH stimulation testing confirmed adrenal insufficiency, although the patient remained asymptomatic, allowing timely hormone substitution to prevent overt adrenal insufficiency.

Most patients with ICI-induced adrenalitis will require lifelong glucocorticoid therapy, with or without mineralocorticoid supplementation. Patient education regarding stress dosing and adrenal crisis prevention is crucial, particularly during intercurrent illness or surgery. Ongoing endocrinology follow-up is essential to monitor replacement adequacy and to adjust therapy over time. Importantly, ^18^F-FDG PET/CT has emerged as a valuable tool for the early detection of ICI-induced adrenalitis, particularly before overt biochemical adrenal insufficiency develops. Typical findings include bilateral, symmetric, and diffusely increased FDG uptake, often accompanied by slight glandular enlargement with preservation of adrenal shape. This imaging pattern contrasts with adrenal metastases, which are more likely to appear as asymmetric nodular enlargements with focal or heterogeneous uptake [43]. The distinctive imaging phenotype of immunotherapy-induced adrenalitis enables clinicians to differentiate inflammatory involvement from metastatic disease, thus guiding early endocrine assessment and the timely initiation of hormone replacement therapy. Early intervention can prevent adrenal crisis, reduce treatment-related morbidity, and avoid unnecessary interruptions of effective oncologic therapies (Figure 4).

### 3.4. Immunotherapy-Induced Pancreatitits and Diabetes Mellitus

**Figure 5:**
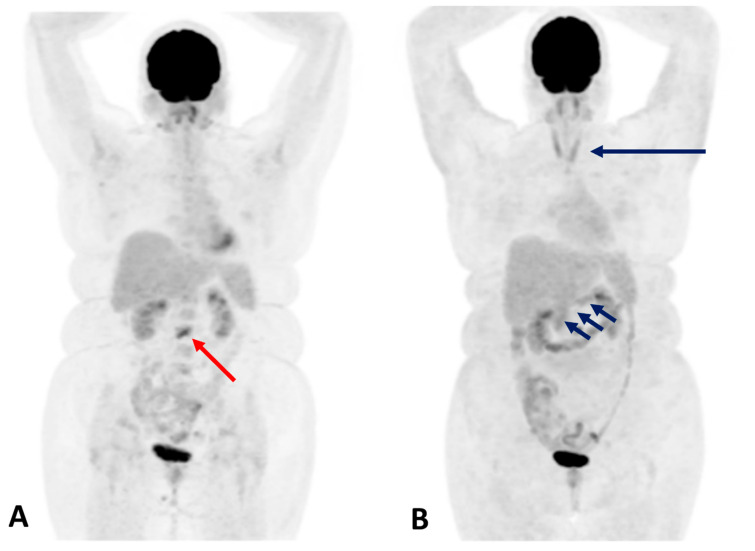
A 53-year-old woman with non-small cell lung cancer underwent surgery, chemotherapy, and radiation therapy for metastatic disease. She subsequently received nivolumab for metastatic progression, most prominently affecting the L2 vertebral body ((**A**), red arrow). Pre-immunotherapy FDG PET/CT demonstrated no abnormal uptake in the pancreas and thyroid. After four months of therapy, she developed abdominal pain and elevated serum amylase (145 UI/L, normal range: 28–100 UI/L). Follow-up ^18^F-FDG PET/CT revealed stable skeletal metastases and new diffuse increased uptake within both the pancreas and thyroid gland ((**B**), blue arrows), consistent with immune-related pancreatitis and thyroiditis. Notably, TSH levels remained within normal limits. Following the temporary discontinuation of immunotherapy and conservative management, the patient’s symptoms improved, and serum amylase levels normalized. Sequential ^18^F-FDG PET/CT imaging can monitor response to corticosteroid therapy, with a decreasing uptake trend indicating resolution of immune-mediated pancreatitis. Importantly, focal intense pancreatic uptake may mimic metastatic disease, underscoring the necessity for careful interpretation within the broader clinical, biochemical, and imaging context [49]. Recognizing characteristic patterns of diffuse or focal ^18^F-FDG uptake in patients receiving ICIs is essential to distinguish immune-mediated pancreatitis from neoplastic or infectious processes, thereby enabling prompt endocrine and gastroenterological evaluation and optimal management of immunotherapy.

Immune checkpoint inhibitor-induced diabetes mellitus (ICI-DM) is a rare but potentially life-threatening endocrine adverse event (EAE). This condition results from immune-mediated destruction of pancreatic β-cells, leading to insulin-dependent diabetes. The pathogenesis is thought to be primarily T-cell driven, reflecting a breakdown in self-tolerance triggered by ICIs. Although clinically similar to type 1 diabetes mellitus (T1DM), ICI-DM frequently lacks classical islet autoantibodies, distinguishing it immunologically from autoimmune T1DM. ICI-DM typically presents abruptly, often after several cycles of therapy, although onset following a single ICI dose has been documented. Characteristic features include severe hyperglycemia, low or undetectable C-peptide levels, and, in many cases, diabetic ketoacidosis (DKA) at initial presentation. The estimated incidence is 0.2–1% of patients receiving ICIs [44]. Given the irreversible loss of β-cell function, early detection is critical. Once diagnosed, lifelong insulin therapy is mandatory, and patients should receive comprehensive diabetes education and close endocrinologic follow-up. Beyond endocrine β-cell injury, ICIs can also trigger immune-mediated inflammation of the exocrine pancreas, leading to autoimmune pancreatitis or exocrine pancreatic insufficiency [45,46]. Immunotherapy-induced pancreatitis, although uncommon (incidence ~0.5 to 4.0%), is a recognized immune-related adverse event (irAE) resulting from T-cell and macrophage infiltration into pancreatic tissue. The clinical spectrum ranges from asymptomatic hyperamylasemia to abdominal pain and, in rare instances, severe acute pancreatitis. Unlike classical pancreatitis, immune-mediated cases are not typically associated with gallstones or alcohol but mirror the pathology of autoimmune pancreatitis. Asymptomatic elevations of pancreatic enzymes do not normally require treatment modification and can be managed conservatively with active surveillance. However, moderate-to-severe pancreatitis warrants temporary interruption of ICI therapy and initiation of glucocorticoid treatment (e.g., prednisone 0.5–1 mg/kg/day), followed by a gradual taper as symptoms improve. In refractory cases, additional immunosuppressive agents may be required. Radiologically, CT and MRI findings resemble those of acute pancreatitis of other etiologies and may include diffuse or focal pancreatic enlargement, peripancreatic fat stranding, and, in severe cases, areas of necrosis with delayed contrast enhancement. In mild or subclinical cases, imaging abnormalities may be minimal or absent. ^18^F-FDG PET/CT has proven useful in detecting subclinical pancreatic inflammation, often revealing diffuse or focal increased FDG uptake with or without pancreatic enlargement, even in patients without clinical symptoms [45,47,48]. These findings can precede or coincide with biochemical abnormalities and guide the decision for closer monitoring or intervention. Moreover, recognizing this uptake pattern may help distinguish immune-mediated pancreatitis from pancreatic metastases, which typically show focal, nodular uptake and may involve adjacent structures. The integration of PET/CT imaging findings with biochemical and clinical data enhances the early diagnosis and optimal management of ICI-associated pancreatic irAEs, minimizing morbidity and supporting the continuation of potentially life-saving immunotherapy (Figure 5).

## 4. Pros and Cons

The use of ^18^F-FDG PET/CT has become increasingly relevant in the evaluation of immune-related endocrine adverse events (irAEs) in patients undergoing TKI therapy and immunotherapy. While this imaging modality offers several advantages, including early detection and whole-body assessment, it also presents certain limitations that impact its clinical utility. Table 5 summarizes the key benefits and drawbacks of ^18^F-FDG PET/CT in the context of detecting endocrine adverse effects associated with immunotherapy.

## 5. Conclusions

The advent of immune checkpoint inhibitors and targeted therapies has revolutionized cancer treatment, substantially improving survival across diverse malignancies. However, these therapeutic advances have introduced a spectrum of endocrine immune-related adverse events (irAEs), including thyroiditis, hypophysitis, adrenalitis, and pancreatitis, which can significantly impact patient management and quality of life if not promptly recognized.

In this context, ^18^F-FDG PET/CT, a cornerstone in oncological staging and treatment monitoring, has emerged as a valuable tool for the early detection and monitoring of endocrine irAEs. Increased ^18^F-FDG uptake in endocrine organs often precedes or coincides with biochemical abnormalities and clinical symptoms of dysfunction. Typical imaging patterns include the following:

- Thyroiditis: Diffuse or heterogeneous increased thyroid uptake, often corresponding to a transition from thyrotoxicosis to hypothyroidism. Focal uptake requires careful evaluation for nodular disease or metastasis.
- Hypophysitis: Homogeneous or asymmetric pituitary uptake associated with symptoms such as headache, visual disturbances, and hypopituitarism.
- Adrenalitis: Symmetric, diffuse adrenal uptake with slight gland enlargement, frequently accompanied by adrenal insufficiency—a potentially life-threatening condition.
- Pancreatitis: Diffuse or focal pancreatic uptake correlating with asymptomatic enzyme elevation or clinical pancreatitis, necessitating differentiation from metastatic disease.

Recognizing these imaging patterns alongside biochemical and clinical assessments is critical for timely diagnosis, optimized management, and prevention of severe endocrine complications in patients undergoing immunotherapy or targeted therapy. Interventions may include hormone replacement or immunosuppressive treatment.

Interestingly, the occurrence of endocrine irAEs and their corresponding imaging findings has been increasingly associated with a more vigorous anti-tumor immune response. Emerging data suggest that these events may correlate with improved oncologic outcomes, reflecting effective systemic immune activation.

Thus, ^18^F-FDG PET/CT extends beyond its traditional role in cancer staging and treatment response assessment, serving as a non-invasive biomarker of immune activation and a valuable tool for the early detection of endocrine irAEs. Accurate interpretation of these findings requires close multidisciplinary collaboration among oncologists, endocrinologists, and nuclear medicine specialists to distinguish immune-mediated inflammation from metastatic disease and to guide timely, personalized management. This integrated approach is essential to optimize cancer therapy outcomes while minimizing the impact of endocrine complications.

## Figures and Tables

**Table 1:** Endocrine adverse events by treatment modalities.

Treatment Type	Endocrine Adverse Events	Prevalence (%)
PD-1/PD-L1 inhibitors	Thyroiditis, hypothyroidism, adrenalitis, diabetes	10–35%
CTLA-4 inhibitors	Hypophysitis, adrenalitis, diabetes	5–10%
Combined ICIs (PD-1 + CTLA-4)	Hypophysitis, multi-endocrine events	30–50%
Pelvic radiotherapy	Gonadal failure, infertility	40–60%
Aromatase inhibitors	Osteoporosis, hypothyroidism	10–30%
Androgen deprivation therapy	Hypogonadism, osteoporosis, metabolic syndrome	50–70%
Tyrosine kinase inhibitors	Hypothyroidism, thyroiditis, Hyperglycemia, Hypoglycemia,	40–70% 15–40%
mTOR inhibitors	Hyperglycemia, insulin resistance, dyslipidemia	10–25%
Chemotherapy (alkylating agents)	Ovarian/testicular failure	30–60%

Legend: CTLA-4, Cytotoxic T-Lymphocyte Antigen 4; mTOR, mechanistic Target of Rapamycin; PD-1/PD-L1, programmed cell death protein-1/programmed death-ligand-1.

**Table 2:** Endocrine adverse effects by tumor type and treatment.

Tumor Type	Primary Treatment	Common Endocrine AEs	Estimated Prevalence (%)
Non-small cell lung cancer	PD-1/PD-L1 inhibitors	Hypothyroidism,	15–35%
		Adrenalitis	<2%
		Diabetes	<1%
Melanoma	CTLA-4 + PD-1 inhibitors	Thyroiditis	25–40%
		Hypophysitis	~10%
		Diabetes	Rare
Breast cancer	AIs, GnRH analogs, and chemotherapy	Ovarian failure	20–50%
		Osteoporosis	5–25%
Prostate cancer	ADT (GnRH agonists/antagonists)	Hypogonadism	>90%
		Osteoporosis	50–70%
		Insulin resistance	30–50%
Renal cell carcinoma	TKIs (e.g., sunitinib)	Hypothyroidism	50–70%
Head/Neck cancer	External beam radiation therapy	Primary hypothyroidism	20–50% (hypothyroidism)
Brain tumors (Pediatric/Adult)	Cranial irradiation	Hypopituitarism -central hypothyroidism, -central hypogonadism -central adrenal failure -GH deficit	30–70%
Hematologic malignancies	Chemotherapy, TBI, HSCT	Gonadal failure	40–60% (gonadal failure),
		GH/TSH/ACTH deficiency	30–50% (HPA axis)
Triple-negative breast cancer	PD-L1 inhibitors (e.g., atezolizumab)	Hypothyroidism,	10–20%
		adrenalitis (rare)	<1%

Legend: ACTH, adrenocorticotropic hormone; ADT, androgen deprivation therapy; AIs, aromatase inhibitors; CTLA-4, Cytotoxic T-Lymphocyte Antigen 4; GnRH, gonadotropin-releasing hormone; GH, growth hormone; HSCT, hematopoietic stem cell transplantation; PD-1/PD-L1, programmed cell death protein-1/programmed death-ligand-1; TSH, thyroid-stimulating hormone; TBI, total body irradiation; TKIs, Tyrosine Kinase Inhibitors.

**Table 3:** Different causes of increased ^18^F-FDG uptake in endocrine glands.

Organ/Gland	Prevalence (PET Studies)	Causes/Etiologies
Thyroid	~0.1 to 4.5 % (mean ~1.6 to 1.9 %)	Mainly autoimmune thyroiditis (e.g., Hashimoto’s), less often Graves’; associated with risk of evolving hypo- or hyperthyroidism
Pituitary	Extremely rare: ~0.073 % focal uptake, but truly diffuse pattern not well characterized	Mostly pituitary adenomas, hypophysitis, metastases, Langerhans cell histiocytosis
Adrenal glands	Diffuse bilateral adrenal uptake is uncommon (studies focus on focal/incidental unilateral lesions: ~7.6 % of scans report adrenal uptake)	Focal adrenal uptake more often: benign adenomas, metastases, adrenal carcinoma, pheochromocytoma; truly diffuse uptake likely physiologic or inflammatory changes, but rare
Pancreas	Diffuse pancreatic uptake is not well described in the literature; usually, only focal uptake is noted (pancreatic inflammation, neoplasm)	Diffuse uptake is likely physiologic or related to inflammatory changes (e.g., pancreatitis), but little formal data exist.

**Table 4:** ^18^F-FDG uptake pattern, imaging features, and clinical correlates of EAEs.

Gland	FDG Uptake Pattern	Associated EAE	Imaging Features	Clinical Presentation
Thyroid	Diffuse increased FDG uptake	Autoimmune thyroiditis (most common irAE)	Symmetrical, homogeneous thyroid uptake; often associated with thyroid atrophy in late stages	Often presents as painless thyroiditis, progressing to hypothyroidism; elevated TPO antibodies are common. May present as transient hyperthyroidism followed by hypothyroidism (biphasic course)
Pituitary	Diffuse or focal increased uptake in the sella turcica	Hypophysitis (especially with anti-CTLA-4)	Mild-to-moderate diffuse uptake; pituitary may be enlarged; can mimic pituitary adenoma	Symptoms: headache, fatigue, nausea, visual changes; lab: ↓ACTH, ↓TSH, ↓LH/FSH, ↓cortisol
Adrenal glands	Mild diffuse bilateral uptake (rarely focal)	Adrenalitis (immune-related adrenal insufficiency)	Bilateral symmetric uptake or volume loss (if late phase); often subtle	Primary adrenal insufficiency: ↓cortisol, ↑ACTH, fatigue, hypotension
Pancreas	Diffuse increased uptake	Immune-related pancreatitis	Pancreatic enlargement, homogeneous increased uptake	Elevated lipase/amylase; symptoms may be absent or include abdominal pain; may require steroids

Legend: ↓, decreased; ↑, increased; FDG, fluorodexossiglucose; TPO, thyroperoxidase; ACTH, adrenocorticotropic hormone; TSH, thyroid-stimulating hormone; LH, luteinizing hormone; FSH, follicle-stimulating hormone.

**Table 5:** Advantages and limitations of ^18^F-FDG PET/CT in EAEs.

Pros	
Advantages	Description
Incidental early detection	PET/CT is often performed for oncologic staging/restaging, allowing opportunistic identification of endocrine AEs even before symptoms occur.
Whole-body assessment	Enables simultaneous evaluation of multiple endocrine organs (thyroid, adrenal, pituitary, pancreas). Useful in systemic immune-related events.
Non-invasive functional imaging	Highlights metabolic changes (inflammation, hyperactivity) before structural changes appear on CT or MRI.
Correlates with inflammation	FDG uptake often corresponds to immune-related inflammation, helping differentiate autoimmune from metastatic or degenerative causes
Potential prognostic relevance	Immune-related AEs, visible on PET, may correlate with better response to immunotherapy (ongoing area of research).
**Cons**	
Limitations	Description
Not specific or diagnostic	FDG uptake is nonspecific: cannot distinguish between inflammation, infection, or malignancy without clinical/lab correlation.
Low sensitivity for some AEs	Many endocrine AEs are biochemically silent or not metabolically active enough to be detected (e.g., central hypothyroidism).
Not routinely indicated	PET/CT is not recommended for diagnosis or follow-up of endocrine AEs—labs and clinical signs usually monitor these.
Risk of overinterpretation	Incidental uptake may lead to unnecessary testing or anxiety if not carefully contextualized.
Radiation and cost	Repeated PET scans have radiation exposure and financial costs, not justifiable solely for AE detection.

## Data Availability

Not applicable.
